# The Effect of Probable COVID-19 Infection on the National Football League Players’ Performance and Endurance During the 2020 Season

**DOI:** 10.7759/cureus.35821

**Published:** 2023-03-06

**Authors:** Clayton R Walker, J Christian Belisario, Benjamin Abramoff

**Affiliations:** 1 Physical Medicine and Rehabilitation, Hospital of the University of Pennsylvania, Philadelphia, USA; 2 Physical Medicine and Rehabilitation, University of Pennsylvania, Philadelphia, USA

**Keywords:** physical medicine and rehabilitation (pm&r), sports medicine, performance, endurance, athletes, nfl, football, covid-19

## Abstract

Objective

The objective of this study was to assess whether the National Football League (NFL) players with probable coronavirus disease 2019 (COVID-19) during the 2020 season experienced a decline in athletic performance and endurance.

Methods

All players who were listed on the NFL’s COVID-19 Injury Reserve (COVID-IR) list were screened for inclusion. Players were included in the study if they had spent ≥10 days on the COVID-19 IR list (which indicated a positive PCR test based on the NFL COVID-19 policies), had played in at least two games before and after going on the IR list, and primarily played an offensive or defensive position. The mean number of snaps played per game and Pro Football Focus (PFF) score per game were collected for each athlete, which served as surrogate measures of endurance and performance, respectively. The results were analyzed with players grouped by position, and then all players grouped as a whole. Within-group comparisons were performed via t-tests.

Results

A total of 78 players met the criteria for inclusion in the study. The overall mean PFF score pre-COVID-19 infection was 62.15 (SD: 6.93), while it was 61.73 (SD: 7.42) post-COVID-19 infection, showing a decrease of 0.42 after infection (n=78, p=0.33). The mean number of snaps played per game pre-COVID-19 infection was 38.99 (SD: 16.46) while it was 38.10 (SD: 17.05) post-COVID-19 infection, showing a decrease of 0.89 after infection (n=78, p=0.30). When grouped by position, statistically significant differences were seen with Defensive Backs’ mean snaps played per game decreasing by 18.30 (n=6, p=0.03) and Defensive Linemen’s mean PFF score decreasing by 3.77 points (n=21, p=0.03).

Conclusion

Based on our findings, COVID-19 infection negatively impacted endurance in Defensive Backs, and performance in Defensive Linemen. However, there was inconclusive evidence to show whether COVID-19 infection negatively impacted other positions when analyzed separately or all positions when analyzed together. Further studies with more participants are needed to fully assess the effects of COVID-19 on performance and endurance in elite athletes.

## Introduction

As the world struggled to balance normalcy with safety amidst the coronavirus disease 2019 (COVID-19) pandemic, many professional sports leagues reopened in the United States prior to vaccines against the disease becoming available. One of the earliest examples, the National Football League (NFL), was able to complete the 2020-2021 season after implementing rigorous testing and isolation protocols, including the administration of over 600,000 COVID-19 tests over the six-month season [1].

Through these protocols, the NFL was generally able to mitigate large outbreaks, although individual players still tested positive for the virus. A recent meta-analysis found that acute symptoms are mild or absent in ~94% of athletes, but 3.8-17% will go on to develop persistent symptoms that may be detrimental to their performance [2]. This is in line with anecdotal reports of some NFL players experiencing a longer duration of symptoms leading to diminished on-field performance. Myles Garrett, a two-time NFL All-Pro Defensive End for the Cleveland Browns, was outspoken about his post-COVID-19 sequela. He was on the COVID-19 Injury Reserve (COVID-IR) list for 11 days. Following this, he reported that he felt he was operating at 50% and that fatigue prevented him from performing at his best for extended periods of time [3]. Similar stories from other notable professional athletes have been reported, including Jayson Tatum, a three-time National Basketball Association (NBA) All-Star who currently plays for the Boston Celtics, and Yoan Moncada who plays second base for the Chicago White Sox of Major League Baseball (MLB) [4,5].

The objective of this study was to evaluate the performance of NFL players before and after a presumed positive test for COVID-19 during the 2020-2021 NFL season.

## Materials and methods

Study design

We adopted a retrospective single-arm cohort study design, using individual player data to compare performance and endurance pre-COVID-19 infection to performance and endurance post-COVID-19 infection. The study was deemed exempt by the University of Pennsylvania Institutional Review Board.

Participants

During the 2020-2021 season, the NFL created a COVID-IR list. This list included players who tested positive or had close-contact exposure to someone with COVID-19. Following a high-risk exposure, a player could return to team activities after having two negative tests within 24 hours. Following a positive test, an asymptomatic player could return to team activities after 10 days since the initial positive test or if he tested negative twice at least 24 hours apart. If the player was symptomatic, he could return to team activities after 10 days from the time symptoms first appeared and after 24 hours since he last experienced symptoms [6]. For the purposes of this study, a player was considered to have a COVID-19 infection if they were placed on the COVID-19 IR list during the 2020-2021 season and were on the list for 10 days or more. Since vaccines for COVID-19 were not available during the study period, all participants were considered unvaccinated.

To exclude players who were placed on the COVID-19 IR list due to high-risk exposure, we excluded any player who was on the COVID-19 IR list for less than 10 days. We also excluded players who played in fewer than two games either before testing positive or after they returned, to allow for averaging of their performance and endurance across multiple games. We also excluded Special Teams players due to the limited data available to determine on-field performance.

All NFL players listed on the NFL’s COVID-IR list during the 2020-2021 season were screened for inclusion.

Data collection and analysis

To assess athletic performance, we compared the mean Pro Football Focus (PFF) score per game pre- and post-COVID-19 infection. PFF is a commonly used grading system that evaluates individual players based on their overall performance in a game. To calculate a player’s PFF score, a panel of professional analysts assigns each player a score from -2 to 2 in 0.5 increments for every play that they are on the field for with 0 being the average or “expected” score. A senior analyst will review all the grades, finalize them, then convert the plus-minus grades to an overall score for the game, ranging from 0 to 100, with 0 being the worst and 100 being the best. PFF evaluates player performance in a standardized manner across all positions, allowing for direct comparison [7]. Overall game scores were collected for every player who met the inclusion criteria. These scores were then averaged pre- and post-COVID-19 infection for comparison.

The publicly available number of “snaps” (plays) that each player participated in was used as a proxy of player endurance. The overall number of “snaps” that players participated in per game was collected for every player who met the inclusion criteria. The number of snaps played per game was then averaged pre- and post-COVID-19 infection for comparison.

Analysis was done for the whole group and by position. Within-group comparisons were performed using t-tests. A p-value of less than 0.05 was considered statistically significant.

## Results

A total of 368 players were placed on the COVID-IR list during the season due to high-risk exposures or a positive PCR test; 78 of the 368 players met the criteria for probable COVID-19 infection and were included in our analysis. The overall mean PFF score pre-COVID-19 infection was 62.15 (SD: 6.93), while it was 61.73 (SD: 7.42) post-COVID-19 infection, showing a decrease of 0.42 after infection (n=78, p=0.33, range: -20.55 to 18.18). The mean number of snaps played per game pre-COVID-19 infection was 38.99 (SD: 16.46), while it was 38.10 (SD: 17.05) post-COVID-19 infection, showing a decrease of 0.89 after infection (n=78, p=0.30, range: -48.83 to 49.68).

When grouped by position, a statistically significant decrease in the mean PFF score was seen among Defensive Linemen (mean decrease: 3.77, n=21, p=0.03). A decrease in mean PFF score was also seen among Tight Ends (mean decrease: 4.12, n=6, p=0.10) and Offensive Linemen (mean decrease: 0.43, n=10, p=0.42). An increase in mean PFF score was seen in Quarterbacks (mean increase: 3.02, n=3, p=0.30), Wide Receivers (mean increase: 0.40, n=12, p=0.42), Defensive Backs (mean increase: 0.89, n=6, p=0.37), Linebackers (mean increase: 2.49, n=10, p=0.27), and Running Backs (mean increase: 2.94, n=11, p=0.07) (Table 1).

**Table 1:** Pro Football Focus scores pre- and post-COVID-19 infection *Indicates statistical significance DB: Defensive Back; DL: Defensive Lineman; LB: Linebacker; OL: Offensive Lineman; PFF: Pro Football Focus; QB: Quarterback; TE: Tight End; WR: Wide Receiver

Position	N	Pre-COVID-19 mean PFF score	Post-COVID-19 mean PFF score	Change	P-value
QB	3	65.01	68.03	3.02	0.30
WR	12	63.57	63.97	0.40	0.42
DB	6	57.23	58.12	0.89	0.37
RB	11	61.24	64.18	2.94	0.07
LB	10	57.62	60.11	2.49	0.27
OL	10	62.94	62.51	-0.43	0.42
DL	21	64.09	60.32	-3.77	0.03*
TE	6	63.13	59.01	-4.12	0.10
Whole group	78	62.15	61.73	-0.42	0.33

When looking at mean snaps per game, a statistically significant decrease was seen among Defensive Backs (mean decrease: 18.30, n=6, p=0.03). A decrease in mean snaps per game was also seen in Wide Receivers (mean decrease: 2.07, n=12, p=0.21), Offensive Linemen (mean decrease: 3.67, n=10, p=0.32), Tight Ends (mean decrease: 2.89, n=6, p=0.30). An increase in the mean snaps per game was seen in Quarterbacks (mean increase: 3.29, n=3, p=0.41), Running Backs (mean increase: 0.86, n=11, p=0.36), Linebackers (mean increase: 5.51, n=10, p=0.24), and Defensive Linemen (mean increase: 1.28, n=21, p=0.23) (Table 2).

**Table 2:** Snap count pre- and post-COVID-19 infection *Indicates statistical significance DB: Defensive Back; DL: Defensive Lineman; LB: Linebacker; OL: Offensive Lineman; PFF: Pro Football Focus; QB: Quarterback; TE: Tight End; WR: Wide Receiver

Position	N	Pre-COVID-19 mean snaps	Post-COVID-19 mean snaps	Change	P-value
QB	3	56.83	60.12	3.29	0.41
WR	12	37.03	34.95	-2.07	0.21
DB	6	43.86	25.57	-18.30	0.03*
RB	11	30.48	31.34	0.86	0.36
LB	10	44.54	50.05	5.51	0.24
OL	10	41.16	37.50	-3.67	0.32
DL	21	36.87	38.14	1.28	0.23
TE	6	40.10	37.21	-2.89	0.30
Whole group	78	38.99	38.10	-0.89	0.30

Post hoc analysis was done by comparing player age, BMI, and time spent on the COVID-IR list with changes in PFF score and changes in the mean number of snaps played. A comparison of age and BMI with the time spent on the COVID-IR list was also done. No statistically significant correlations were found (Tables 3, 4 in the Appendix).

## Discussion

COVID-19 is a viral illness that infiltrates the body via the respiratory tract [8]. It can cause direct cellular damage to the lung parenchyma as well as systemic damage by triggering a cytokine storm [9]. Through the systemic inflammatory process, COVID-19 can cause myocarditis, pericarditis, or direct myocardial injury [10]. The data regarding the prevalence of myocardial abnormalities after COVID-19 infection in the athlete population varies considerably but is thought to be around 5% [2]. Due to the concern for sudden cardiac death, extensive screening is recommended by the American College of Cardiology for athletes with severe or persistent symptoms prior to returning to sport [11]. Even after being medically cleared to return to sport, some athletes feel lingering symptoms that subjectively impact their performance, which this study attempts to quantify [1-6,12].

Although a decrease in the mean PFF score and the mean number of snaps played after the COVID-19 infection was found when looking at all players, these were neither statically significant nor very large in magnitude. However, when analyzed by position, there was a statistically significant decrease in the mean number of snaps played by Defensive Backs and a statistically significant decrease in Defensive Linemen’s mean PFF score. Other positions also showed trends that were approaching statistical significance (e.g., Tight Ends’ decrease in PFF score and Running Backs’ increase in PFF score).

The variability in outcomes may be due to the wide range of symptoms experienced by those with COVID-19 infection and the heterogeneity of the positions played by the athletes and their unique physical demands. For example, Defensive Backs experience fewer high-energy collisions but run more than any other position on defense, covering about 3 miles per game [13,14]. Given the endurance component of their position compared to other defensive positions, this may explain why this group saw a statistically significant decrease in the mean number of snaps per game after returning from COVID-19 infection while other positions did not. Defensive Linemen, alternatively, had a statically significant decrease in their mean PFF score after returning from COVID-19. This group had the highest BMI of all the defensive players, at 36.31 kg/m^2^. Obesity has been correlated with worse COVID-19 symptoms, which may explain this trend [15]. Running Backs, who sustain the greatest number of severe impacts (>10 G force) per game, had an increase in mean PFF score that approached statical significance [13]. We hypothesize that the time away from sport allowed them to better recover from the intense physical demands of their position and thus perform better upon returning.

Limitations

There are numerous confounding factors that exist within an NFL season that may impact an individual player’s PFF score or number of snaps played, none of which were able to be analyzed in our study. A few examples of confounding factors are injuries to the players or their teammates, trades between teams involving the players or their teammates, different game strategies used on a weekly basis, changes in the difficulty of the opposition on a weekly basis, and differences in refereeing by in-game officials. Our study was further limited by the small number of players that met the inclusion criteria for each position and by not having access to NFL testing data and official medical history for the players. Hence, COVID-19-positive cases were deduced using the NFL isolation protocol and the amount of time players spent on the COVID-IR list, which may have resulted in excluding some true positive cases and failing to differentiate asymptomatic cases from symptomatic cases. Lastly, it was impossible to distinguish performance changes due to COVID-19 infection vs. changes due to the athlete having several days of rest before returning to sport.

## Conclusions

Based on our findings, probable COVID-19 infection negatively impacted endurance among Defensive Backs, and performance among Defensive Linemen. However, the evidence was inconclusive as to whether COVID-19 infection negatively impacted other positions when analyzed separately or all positions when analyzed together. Further studies with more participants are warranted to gain deeper insights into the effects of COVID-19 on performance and endurance among professional American football athletes.

## Appendices

**Table 3:** Demographic data by position DB: Defensive Back; DL: Defensive Lineman; COVID-IR: COVID-19 Injury Reserve; LB: Linebacker; OL: Offensive Lineman; QB: Quarterback; TE: Tight End; WR: Wide Receiver

	Position	N	Mean age (years)	Similar to:	Mean BMI (kg/m^2^)	Similar to:	Mean time spent on COVID-IR list (days)	Similar to:
Positions	QB	3	29.33	All	28.68	None	12	All
	WR	12	27.67	All	26.52	DB	13.55	All
	DB	6	26.80	All	27.14	WR	13.4	All
	RB	11	25.91	All	31.60	LB, TE	14.82	All
	LB	10	27.10	All	30.94	TE, RB	12.2	All
	OL	10	26.60	All	37.80	DL	13.7	All
	DL	21	26.81	All	36.31	OL	12.1	All
	TE	6	25.83	All	30.59	RB, LB	12	All
	Whole group	78	26.85	-	32.32	-	12.95	-

**Table 4:** Correlation coefficients comparing different variables Correlation coefficients were calculated for changes in snaps and changes in Pro Football Focus (PFF) score compared to Age, BMI, and time on COVID-19 Injury Reserve (COVID-IR) list, and for Age and BMI compared to time on COVID-IR List

	Age	BMI	Time on COVID-IR list
Change in snaps	0.08	0.10	0.12
Change in PFF score	0.08	-0.11	0.02
Age	-	-	-0.04
BMI	-	-	0.06
